# Wastewater Surveillance for SARS-CoV-2 at Long-Term Care Facilities: Mixed Methods Evaluation

**DOI:** 10.2196/44657

**Published:** 2023-08-29

**Authors:** James W Keck, Jess Lindner, Matthew Liversedge, Blazan Mijatovic, Cullen Olsson, William Strike, Anni Noble, Reuben Adatorwovor, Parker Lacy, Ted Smith, Scott M Berry

**Affiliations:** 1 Department of Family & Community Medicine University of Kentucky Lexington, KY United States; 2 College of Medicine - Northern Kentucky Campus University of Kentucky Highland Heights, KY United States; 3 Department of Family and Community Medicine University of Kentucky Lexington, KY United States; 4 Department of Biomedical Engineering University of Kentucky Lexington, KY United States; 5 Department of Mechanical Engineering University of Kentucky Lexington, KY United States; 6 Department of Biostatistics University of Kentucky Lexington, KY United States; 7 Trilogy Health Services Louisville, KY United States; 8 Christina Lee Brown Envirome Institute University of Louisville Louisville, KY United States

**Keywords:** wastewater surveillance, wastewater-based epidemiology, evaluation, long-term care facility, COVID-19, SARS-CoV-2

## Abstract

**Background:**

Wastewater surveillance provided early indication of COVID-19 in US municipalities. Residents of long-term care facilities (LTCFs) experienced disproportionate morbidity and mortality early in the COVID-19 pandemic. We implemented LTCF building-level wastewater surveillance for SARS-CoV-2 at 6 facilities in Kentucky to provide early warning of SARS-CoV-2 in populations considered vulnerable.

**Objective:**

This study aims to evaluate the performance of wastewater surveillance for SARS-CoV-2 at LTCFs in Kentucky.

**Methods:**

We conducted a mixed methods evaluation of wastewater surveillance following Centers for Disease Control and Prevention (CDC) guidelines for evaluating public health surveillance systems. Evaluation steps in the CDC guidelines were engaging stakeholders, describing the surveillance system, focusing the evaluation design, gathering credible evidence, and generating conclusions and recommendations. We purposively recruited stakeholders for semistructured interviews and undertook thematic content analysis of interview data. We integrated wastewater, clinical testing, and process data to characterize or calculate 7 surveillance system performance attributes (simplicity, flexibility, data quality, sensitivity and positive predictive value [PPV], timeliness, representativeness, and stability).

**Results:**

We conducted 8 stakeholder interviews. The surveillance system collected wastewater samples (N=811) 2 to 4 times weekly at 6 LTCFs in Kentucky from March 2021 to February 2022. Synthesis of credible evidence indicated variable surveillance performance. Regarding simplicity, surveillance implementation required moderate human resource and technical capacity. Regarding flexibility, the system efficiently adjusted surveillance frequency and demonstrated the ability to detect additional pathogens of interest. Regarding data quality, software identified errors in wastewater sample metadata entry (110/3120, 3.53% of fields), technicians identified polymerase chain reaction data issues (140/7734, 1.81% of reactions), and staff entered all data corrections into a log. Regarding sensitivity and PPV, using routine LTCF SARS-CoV-2 clinical testing results as the *gold standard*, a wastewater SARS-CoV-2 signal of >0 RNA copies/mL was 30.6% (95% CI 24.4%-36.8%) sensitive and 79.7% (95% CI 76.4%-82.9%) specific for a positive clinical test at the LTCF. The PPV of the wastewater signal was 34.8% (95% CI 27.9%-41.7%) at >0 RNA copies/mL and increased to 75% (95% CI 60%-90%) at >250 copies/mL. Regarding timeliness, stakeholders received surveillance data 24 to 72 hours after sample collection, with delayed reporting because of the lack of weekend laboratory staff. Regarding representativeness, stakeholders identified challenges delineating the population contributing to LTCF wastewater because of visitors, unknown staff toileting habits, and the use of adult briefs by some residents preventing their waste from entering the sewer system. Regarding stability, the reoccurring cost to conduct 1 day of wastewater surveillance at 1 facility was approximately US $144.50, which included transportation, labor, and materials expenses.

**Conclusions:**

The LTCF wastewater surveillance system demonstrated mixed performance per CDC criteria. Stakeholders found surveillance feasible and expressed optimism regarding its potential while also recognizing challenges in interpreting and acting on surveillance data.

## Introduction

### Background

Two years into the COVID-19 pandemic, myriad public health challenges remain. Preventing disease in populations considered vulnerable is among the most persistent and pressing issues. Older adults, particularly those living in long-term care facilities (LTCFs), experienced disproportionate morbidity and mortality during the COVID-19 pandemic [1]. An estimated 23% of the COVID-19 deaths in the United States occurred among LTCF residents or staff as of early 2022 [2]. A combination of age-related relative immunosuppression, high prevalence of comorbid conditions, and increased exposure in congregate living settings contributes to a greater burden of infection and mortality among LTCF residents [1].

Early efforts in the pandemic to prevent and mitigate COVID-19 at LTCFs focused on limiting exposure (physical distancing and personal protective equipment) and symptom screening to identify potential cases [3]. There was limited availability of clinical testing; for example, in June 2020, all LTCF staff and residents in Kentucky underwent a single comprehensive round of SARS-CoV-2 testing, and, at the time, it was unclear whether more frequent clinical testing was feasible. We identified wastewater surveillance for SARS-CoV-2 as a promising approach to direct the limited clinical testing resources and potentially mitigate the spread of infection at LTCFs.

Wastewater surveillance analyzes samples of wastewater for the presence of disease markers, such as SARS-CoV-2 RNA [4]. Many individuals who have been infected with SARS-CoV-2 shed the virus in their stool [5], and there is evidence that wastewater viral trends precede clinical case detection by several days [6]. Monitoring wastewater for SARS-CoV-2 RNA can identify previously unknown cases in a population [7] and guide clinical testing and infection prevention activities to mitigate new infections [7,8].

Wastewater surveillance has potential advantages over clinical testing. Foremost, it measures disease presence in a population via a single sample, making it more efficient than individual surveillance [9]. It also has the potential to detect SARS-CoV-2 from asymptomatic individuals who might not otherwise seek clinical testing [6]. It is noninvasive, which means that it may be more acceptable to individuals than repeated clinical testing [10]. Wastewater surveillance may provide more timely information on disease presence and trends than clinical testing owing to the earlier detection of SARS-CoV-2 shed by asymptomatic or presymptomatic individuals in the population [6]. However, there is little evidence to guide the implementation of wastewater surveillance at LTCFs.

### Objectives

We share our experience of implementing wastewater surveillance for SARS-CoV-2 at 6 LTCFs in Kentucky during the second year of the COVID-19 pandemic. We evaluated the LTCF wastewater surveillance according to the framework put forth by the Centers for Disease Control and Prevention (CDC) Guidelines Working Group [11] for evaluating public health surveillance systems.

## Methods

### Evaluation Approach and Manuscript Organization

The surveillance evaluation followed CDC guidelines for evaluating public health surveillance systems [11]. We followed the guidelines’ evaluation steps, including (1) engaging stakeholders, (2) describing the surveillance system, (3) focusing the evaluation design, (4) gathering credible evidence, and (5) generating conclusions and recommendations. We describe step 1 (engaging stakeholders) and how we gathered and analyzed credible evidence (steps 4 and 5) in this *Methods* section. In the *Results* section, we describe the surveillance system (step 2) and share credible evidence (step 4). In accordance with CDC surveillance evaluation guidelines, we gathered evidence on 7 general performance attributes of surveillance systems (simplicity, flexibility, data quality, sensitivity and positive predictive value [PPV], timeliness, representativeness, and stability). We incorporated additional concerns that arose during stakeholder interviews into the evaluation. We provide conclusions and recommendations (step 5) in the *Discussion* section.

### Stakeholder Interviews and Qualitative Analysis

The CDC guidelines identified stakeholder groups potentially relevant to surveillance system evaluation to include public health practitioners, health care providers, data providers and users, representatives of affected communities, governments, and professional and private organizations. The study team identified the following stakeholder groups to focus the evaluation design (step 3) and provide credible evidence (step 4) relevant for the evaluation: LTCF management (encompassing the categories of data provider and user, health care provider, representative of affected community, and private organization), public health practitioners, and wastewater testing field and laboratory staff (another category of data provider). We purposively recruited individuals from these groups based on their involvement with LTCF wastewater surveillance and jurisdictional relationship (eg, local public health department) for semistructured interviews tailored to stakeholder roles. Interview questions asked about LTCF wastewater surveillance usefulness, resources, challenges, communication, concerns, and actions. The semistructured interview guides are presented in Multimedia Appendix 1. Author JL, a medical student not involved in implementing the wastewater surveillance system, conducted the interviews remotely using videoconferencing. Stakeholder interviewees provided written informed consent before the interview; they did not receive compensation for their participation. Each stakeholder was interviewed once, and the interviews lasted 18 to 35 minutes. We recorded and transcribed the interviews for qualitative analysis. We analyzed interview content using the rigorous and accelerated data reduction approach for qualitative analysis [12]. The rigorous and accelerated data reduction method involved identifying key points and moving pertinent quotes or thoughts into a central spreadsheet. After each interview transcript underwent this process, we identified common themes and moved these data into a secondary spreadsheet. The data went through several rounds of this reduction process to isolate the most pertinent ideas and generate representative quotations. We used information from the stakeholder interviews to focus the evaluation design (step 3).

### Other Credible Data Sources

Credible evidence (step 4 of the evaluation process) came from several sources. The project’s LTCF partner shared deidentified staff and resident clinical testing results from the 6 facilities during the wastewater surveillance period. LTCF clinical testing occurred per state, CDC, and Centers for Medicare and Medicaid Services guidance and varied from twice weekly to monthly screening of unvaccinated staff per local disease transmission levels. Wastewater testing data came from the study team. The stakeholder interviews provided qualitative data. Additional data came from the project team’s administrative records.

### Wastewater Surveillance Performance

We calculated the sensitivity and specificity of wastewater surveillance by comparing wastewater positivity with clinical test positivity for SARS-CoV-2 at wastewater thresholds ranging from 0 to 250 copies/mL of SARS-CoV-2 RNA. The study team obtained wastewater measurements using a method developed by our team and described in the *Surveillance System Description* subsection of the *Results* section. We compared the wastewater data with clinical test results during the week that followed a wastewater measurement in a 2×2 contingency table (true positives, true negatives, false positives, and false negatives) at multiple wastewater SARS-CoV-2 threshold values. We then estimated the sensitivity and specificity of wastewater surveillance at each threshold and plotted a receiver operating characteristic curve. To evaluate whether wastewater testing identified SARS-CoV-2 at LTCFs earlier than routine clinical screening, we conducted a lead-lag time correlational analysis. We estimated the correlation between the average wastewater RNA concentration and the number of known SARS-CoV-2 infections at a specific LTCF by shifting the wastewater result 1 to 7 days before and after the clinical test collection date. We calculated the Kendall rank correlation coefficient because this estimator is conservative compared with the Pearson and Spearman correlation coefficients. We used Microsoft Excel for descriptive analyses of administrative data and SAS 9.4 (SAS Institute Inc) to estimate the wastewater surveillance performance metrics of sensitivity and PPV, and the correlation of the wastewater RNA signal with clinical test data.

### Ethics Approval

The University of Kentucky Institutional Review Board reviewed and approved the study protocol (62384).

## Results

### Surveillance System Description

#### Overview

The study team collaborated with Trilogy Health Services, an LTCF organization that manages >100 facilities across the Midwest in the United States. The study team identified 6 LTCFs in Lexington and Louisville, Kentucky, for wastewater surveillance. Proximity to the University of Kentucky campus and the ability to access facility-specific wastewater effluent guided facility site selection. The 6 participating LTCFs each had 67 to 160 residents and 76 to 117 staff. Wastewater sampling began on March 19, 2021, and ended in Louisville on December 16, 2021, and in Lexington on February 23, 2022.

#### Wastewater Collection

The study team visited each LTCF to identify wastewater effluent access points with facility management. For each facility, there was manhole access to a sewer pipe that contained the entirety of the facility’s wastewater effluent and did not contain effluent from neighboring buildings. Field technicians obtained LTCF wastewater effluent samples using autosamplers suspended under the manhole covers that collected 100 mL of wastewater every 20 minutes during a 24-hour period (ie, a composite sample). Rechargeable batteries provided power for the autosamplers. Ice packed around the autosampler jug refrigerated the composite sample to minimize RNA degradation. The technicians collected three to four 24-hour composite samples each week from the Lexington LTCFs and 2 to 3 samples each week from the Louisville LTCFs. Field technicians recorded wastewater composite sample collection date, location, volume, and temperature using tablet computers and a custom REDCap (Research Electronic Data Capture; Vanderbilt University) database. The field technicians transported 250 mL from each 24-hour composite sample to the University of Kentucky in Lexington for analysis. The team collected 811 composite wastewater samples across the 6 facilities during the study period.

#### Laboratory Analysis

Laboratory analysis of the wastewater included (1) heat-mediated viral lysis, (2) nucleic acid extraction using paramagnetic particles with exclusion-based sample extraction [13], and (3) SARS-CoV-2 RNA quantification using CDC-recommended N1 primers and real-time qualitative polymerase chain reaction (RT-qPCR) analysis. Laboratory staff processed 8 aliquots from each sample and reported the average SARS-CoV-2 RNA concentration across the 8 aliquots. Strike et al [14] provide a detailed description of the laboratory method. Laboratory quality control measures included negative RT-qPCR controls, positive RT-qPCR controls, spiked SARS-CoV-2 samples to assess RNA extraction efficiency, and a visual inspection of RT-qPCR readouts for concurrence with automated cycle threshold reads. In addition, the laboratory measured crAssphage DNA concentrations in 2 aliquots from each wastewater composite sample as an indicator of the sample’s fecal load using the same general laboratory method as for SARS-CoV-2 quantification. crAssphage is a human gut bacteriophage that is ubiquitous in human stool at high concentrations.

#### Data Architecture and Communication

The study team developed a custom computer program to join the wastewater sample data stored in the REDCap database with the RT-qPCR wastewater RNA data, which resulted in a text file. The program incorporated multiple data validity checks and generated an error log that triggered study team members to investigate and rectify data quality issues. The team created a web-based password-protected data visualization dashboard that provided up-to-date results of wastewater surveillance to Trilogy management. The study team also shared wastewater surveillance results with Trilogy facility leadership via email and telephone. Trilogy decided how to respond to the wastewater surveillance data, including any enhanced clinical testing of staff or residents.

### Wastewater Surveillance Performance

In accordance with CDC surveillance evaluation guidelines, we present credible evidence from stakeholder interviews (N=8), administrative records, wastewater analysis, and clinical testing results to address 7 general performance attributes of public health surveillance systems.

#### Simplicity: How Cumbersome Was the Process of Collecting Samples, Processing Them, and Sharing Surveillance Data With Stakeholders?

Testing wastewater samples 3 to 4 times per week at 6 facilities across 2 municipalities required 1 full-time laboratory technician, 1 full-time field technician, and 1 part-time field technician. Several part-time student laboratory assistants worked ≤10 hours a week to conduct wastewater laboratory analyses under the supervision of the full-time laboratory technician. The field technicians had no relevant experience at surveillance onset and acquired wastewater collection skills through apprenticeship during several field site visits.

The method of communicating wastewater results evolved over the study. Data sharing transitioned from emails and telephone calls to a web-based data dashboard, complemented by emails and telephone calls for results with perceived urgency. The dashboard displayed levels of SARS-CoV-2 RNA in wastewater by LTCF location and included clinical testing data from Trilogy’s public-facing COVID-19 dashboard.

#### Flexibility: Did Wastewater Surveillance Adapt to the Changing Realities of the Pandemic, and If So, How Much Effort Was Needed, and How Successful Was the Transition?

Wastewater surveillance demonstrated flexibility in sampling frequency and duration, population under surveillance, and pathogens targeted. Initially, the field team collected wastewater samples 3 times weekly (Lexington) and twice weekly (Louisville). The team increased the sampling frequency to 4 times weekly (Lexington) and 3 times weekly (Louisville) to optimize the timely identification of new infections in the LTCF population. In addition, the study team reported that they efficiently resumed wastewater surveillance in January 2022 (after planned completion in December 2021) in Lexington because of concern for infections related to the SARS-CoV-2 Omicron variant in the LTCF population.

Although the focus of the wastewater surveillance system was SARS-CoV-2, the study team piloted testing LTCF wastewater for *Clostridioides difficile*, a pernicious colonic bacterium that can cause severe gastrointestinal illness. The testing of LTCF wastewater identified pathogenic (toxin producing) *C difficile* DNA, suggesting the flexibility of LTCF wastewater surveillance for pathogens beyond SARS-CoV-2.

#### Data Quality: How Complete Were the Wastewater Surveillance Data? How Was Data Quality Assured?

The wastewater surveillance system obtained 24-hour composite wastewater samples from the LTCFs 2 to 4 days per week. In Lexington, wastewater effluent was tested 47.3% (160/338) of the days in the study period. In Louisville facilities, wastewater testing covered 35.8% (98/274) to 37.2% (102/274) of the days in the study period. The subsection describing system stability provides additional information regarding wastewater sample collection issues that may have affected data quality. Of the 811 wastewater samples collected, 31 (3.8%) were not processed owing to reagent shortages (n=21, 68%), processing delays after winter storms (n=9, 29%), and contamination during laboratory extraction (n=1, 3%). Of the 780 samples analyzed for SARS-CoV-2, 40 (5.1%) had no detectable concentration of crAssphage or were not analyzed for crAssphage. The absence of detectable crAssphage in a wastewater sample suggests low or negligible presence of fecal material, which makes the detection of SARS-CoV-2 RNA less likely.

Custom software identified data inconsistencies in the REDCap database and the polymerase chain reaction (PCR) output files. In addition, laboratory personnel visually reviewed PCR curves and data generated by the PCR instrument to identify erroneous machine-estimated results. To maintain data integrity, technicians entered corrections into an Excel workbook. The software applied these corrections to the REDCap data before joining the data with the PCR results. The frequencies of identified data issues are presented in Table 1.

**Table 1:** Frequency of identified quality issues in long-term care facility wastewater surveillance data.

Data source and data quality issue		Values, n (%)
**REDCap^a^ (n=780)**		
	Inaccurate sample ID	89 (11.4)
	Inaccurate location ID	16 (2.1)
	Inaccurate collection method	0 (0)
	Inaccurate sample date	5 (0.6)
**RT-qPCR^b^ (n=7734)**		
	False-positive PCR^c^ value	91 (1.2)
	False-negative PCR value	2 (0)
	Inaccurate PCR value	19 (0.2)

^a^REDCap: Research Electronic Data Capture.

^b^RT-qPCR: real-time qualitative polymerase chain reaction.

^c^PCR: polymerase chain reaction.

#### Sensitivity and PPV: What Proportion of the Time When There Was a Known SARS-CoV-2 Case Was There a Positive Wastewater SARS-CoV-2 Signal? What Was the Probability That a Positive Wastewater Signal Indicated an Active SARS-CoV-2 Infection?

We used Trilogy’s clinical testing data to estimate the performance of wastewater surveillance for detecting SARS-CoV-2 infections. Trilogy primarily used antigen-based SARS-CoV-2 tests. Trilogy tested LTCF staff per CDC and state guidance, which recommended PCR testing of staff who were symptomatic and every-other-week to twice-a-week testing of unvaccinated staff based on COVID-19 activity in the facility’s county. Residents received SARS-CoV-2 testing when symptomatic and twice weekly after the identification of a case in the facility until no new cases were identified for 2 weeks. Trilogy did not require visitors to test.

**Figure 1:**
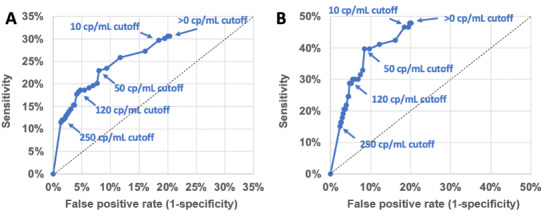
Receiver operating characteristic curves depicting the sensitivity and specificity of wastewater surveillance at varying SARS-CoV-2 wastewater signal thresholds to discriminate the presence of staff and residents with a positive SARS-CoV-2 clinical test at 6 long-term care facilities. Note: panel A includes resident and staff clinical test results; panel B includes only resident clinical test results. cp: copies.

**Figure 2:**
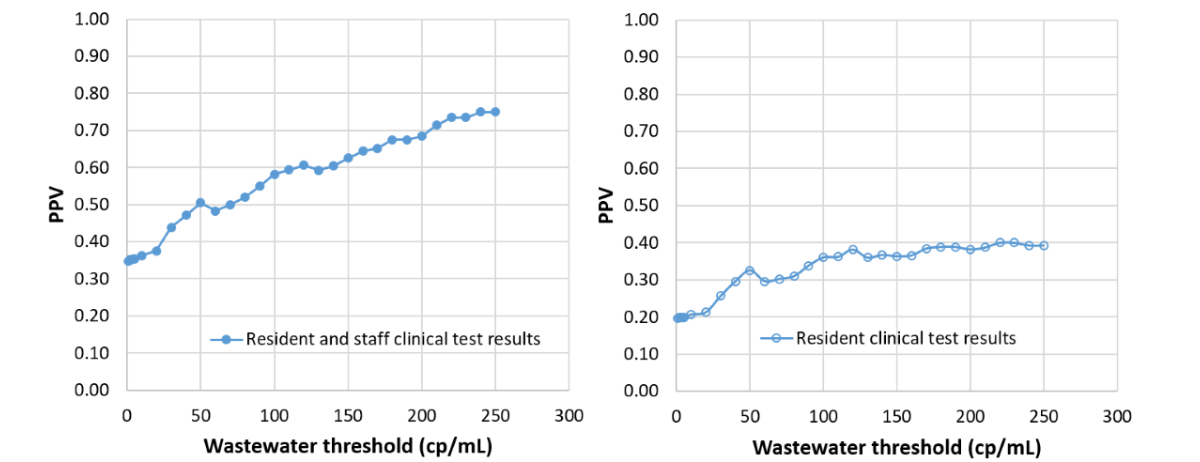
Positive predictive value (PPV) of a SARS-CoV-2 wastewater signal at various threshold values for the presence of a long-term care facility staff member or resident with a positive SARS-CoV-2 clinical test. cp: copies.

Wastewater surveillance sensitivity for detecting the presence of identified clinical cases was 30.6% (95% CI 24.4%-36.8%) using a signal threshold of >0 RNA copies/mL and decreased to 11.5% (95% CI 7.2%-15.8%) with a signal threshold of >250 RNA copies/mL (Figure 1). Wastewater surveillance specificity ranged from 79.7% (95% CI 76.4%-82.9%; >0 RNA copies/mL) to 98% (95% CI 97.7%-99.6%; >250 RNA copies/mL). When limiting clinical test data to residents, wastewater surveillance sensitivity at a signal threshold of >0 RNA copies/mL improved to 48% (95% CI 36.5%-59.4%), with a specificity of 80% (95% CI 77%-82.9%; Figure 1). The PPV of wastewater surveillance ranged from 34.8% (95% CI 27.9%-41.7%) to 75% (95% CI 60%-90%) for wastewater signal thresholds of >0 copies/mL and >250 copies/mL, respectively, when including all clinical data and ranged from 19.7% (95% CI 13.8%-25.5%) to 39.3% (95% CI 21.2%-57.4%) when considering only clinical tests from residents (Figure 2).

Limitations of the clinical testing data likely affected the estimates of wastewater surveillance performance. Rapid antigen test sensitivity is poor in asymptomatic individuals [15], and there was incomplete and variable testing of residents and staff. There was likely underascertainment of SARS-CoV-2 infections in the LTCF population, which would falsely deflate the estimates of wastewater surveillance specificity and PPV and inflate the estimates of sensitivity.

#### Timeliness: How Much Time Elapsed Between Collecting a Wastewater Sample, Analytic Results, and Infection Prevention Action by the Facility? Did Facility-Level Wastewater Surveillance Detect the Presence of SARS-CoV-2 Sooner Than Routine Clinical Testing or Testing Triggered by Symptom Screening?

Wastewater surveillance data were typically available for stakeholders 24 to 72 hours after sample collection. Variation in data timeliness was due primarily to personnel availability for sample processing and data entry. In general, turnaround time was faster for samples collected on Tuesdays, Wednesdays, and Thursdays than for samples collected on Fridays because laboratory staff did not work on weekends. Median turnaround times for results for samples collected on Tuesdays, Wednesdays, and Thursdays were 26, 37, and 28 hours, respectively. For results for samples collected on Fridays, the median turnaround time was 74 hours.

**Figure 3:**
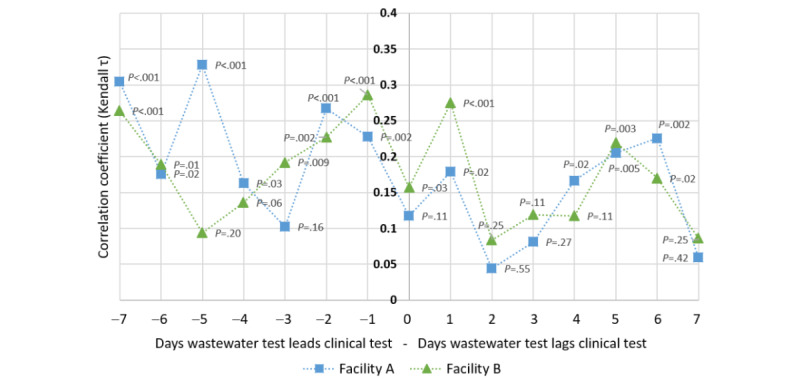
Lagged correlational analysis of wastewater SARS-CoV-2 positivity with SARS-CoV-2 clinical test positivity at 2 long-term care facilities. The x-axis indicates the number of days the wastewater data are offset relative to the clinical data, with negative values indicating the number of days that wastewater data lead, and positive values indicating the number of days that wastewater data lag, the clinical test data.

A lagged correlation analysis between the wastewater signal and clinical case detection showed variable correlation across the 6 facilities. At the 2 Lexington facilities with the greatest number of clinical cases, the wastewater signal correlated temporally with clinical testing results (Figure 3). Although wastewater positivity generally led clinical positivity (rather than lagged), there was not a specific lead time that outperformed all others because significant correlations were seen for lead times ranging from 1 (*P*<.001) to 7 (*P*<.001) days.

#### Representativeness: How Thoroughly Did Wastewater Testing Monitor the Population in the LTCFs?

Wastewater surveillance was representative of the population contributing to the wastewater at LTCFs because it examined building-wide sewer effluent over 24 hours. However, there were important caveats. The LTCF population that potentially contributed to wastewater at the facility was dynamic: there were resident admissions and discharges, staff worked at ≥1 locations, and the LTCFs reinstated resident visitation during the surveillance period. In addition, members of the LTCF population may not have contributed to the sewer effluent because they used toilets outside of the facility (staff) or because of incontinence and the use of adult briefs (residents). As previously mentioned, the inclusion of staff clinical results alongside resident results substantially affected the sensitivity (lower) and PPV (higher) of wastewater surveillance.

During a cluster of 10 identified resident cases at 1 LTCF, the surveillance team noted an intermittently positive wastewater signal. An investigation of this unexpected variability revealed that 6 (60%) of the 10 residents were fully or partially incontinent and using adult briefs. Their feces did not enter the wastewater stream and therefore did not contribute to a measurable SARS-CoV-2 wastewater signal.

#### Stability: What Issues Arose With Surveillance Equipment, Processes, Data Collection, or Reporting, and How Did This Affect Surveillance? How Much Did the System Cost to Operate?

Global supply chain issues owing to the COVID-19 pandemic required flexibility. At the study onset, autosamplers were unavailable, which delayed surveillance implementation by a month. Intermittent vendor shortages of laboratory supplies forced reuse protocols when possible and resulted in a reduction of replicates analyzed in 9.5% (74/780) of the samples. Several environmental and infrastructure factors challenged wastewater collection. Autosampler logs identified periods of low flow in the wastewater effluent stream, which resulted in smaller sample volumes and less representative composite samples at 1 facility in particular. We did not observe clogging of the strainers at the end of the autosampler tubing; however, intermittent clogging may have occurred and contributed to the low flow measured by the autosamplers. The results from the days and locations with smaller composite volumes may not represent conditions from the 24-hour collection period. In addition, sewer architecture resulted in a likely false-positive wastewater signal at 1 LTCF (Textbox 1).

Wastewater composite samples exceeded the goal temperature threshold of 4 °C (for optimal RNA stability) during summer months. Decreasing the composite sample volume (to 6000 mL) and increasing the volume of ice used in the autosamplers increased the frequency with which the composite wastewater samples stayed below the target temperature.

The reoccurring cost to conduct 1 day of wastewater surveillance at an LTCF was approximately US $144.50, or approximately US $18.06 per replicate. This estimate included transportation, labor, and materials based on wastewater testing at 6 sites (Table 2). Additional 1-time equipment expenses, such as laboratory equipment (notably a PCR machine) and autosamplers, contribute to start-up costs for wastewater surveillance. Amortized over the lifetime of the equipment, these expenses will increase per-sample costs by approximately US $5 to US $10. There are potential economies of scale related to labor because there are time efficiencies when obtaining and processing samples from multiple facilities.

**Adapting wastewater sampling to mitigate sewer architecture challenges.**

**Figure 4:**
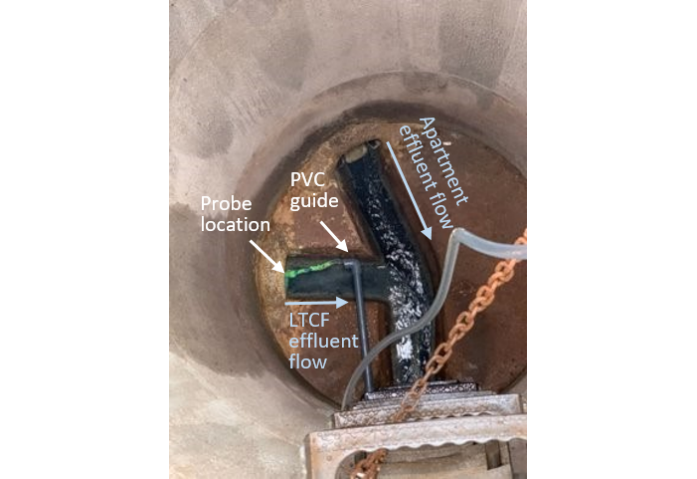
Autosampler probe guide designed to address the sampling issue at a long-term care facility (LTCF). PVC: polyvinyl chloride.

During routine wastewater surveillance, the team detected a strongly positive wastewater signal at a facility with no known COVID-19 infections. This happened on a Friday, and the surveillance team notified long-term care facility (LTCF) leadership shortly before the close of business. Over the subsequent 2 days, the LTCF leadership tested all residents and staff at the facility but did not identify an individual with SARS-CoV-2 infection. LTCF site management shared that there were COVID-19 cases at a neighboring apartment building. An examination of sewer architecture revealed that wastewater effluent from the LTCF and the apartment building converged at our sampling point (Figure 4). Although the field technician placed the autosampler probe into the LTCF wastewater effluent channel, the technician noted intermittent probe migration into the convergent channel. We presume that the strongly positive wastewater signal came from the apartment building. We addressed this sampling issue by designing an autosampler probe guide made using a polyvinyl chloride pipe and rigid wire. This custom device ensured that the probe remained in the channel containing only LTCF wastewater.

**Table 2:** Cost estimates for wastewater surveillance at long-term care facilities by expense category.

	Cost/replicate (US $)	Cost/sample (US $)	Cost per capita^a^/sample (US $)
Reagents	6.47	51.78	0.28
Labor	7.30	58.38	0.31
Transportation	4.29	34.33	0.18
Total	18.06	144.50	0.77

^a^On the basis of the total population (N=1124) of residents and staff at the 6 long-term care facilities at the conclusion of wastewater surveillance.

### Additional Wastewater Surveillance Considerations

Stakeholders mentioned that the relative newness of wastewater surveillance added complexity to surveillance implementation. The collaboration between university researchers and the LTCF organization raised data sharing and privacy issues related to resident and staff COVID-19 testing results. A formal data use agreement clarified expectations.

All interviewed stakeholders raised the issue of how best to interpret, and respond to, the wastewater data. LTCF and local health department leadership expressed a desire for a clear course of action. They asked questions about the strength of a wastewater signal that should trigger clinical testing and about how wastewater RNA concentrations correlated to the numbers of infections at the facility. Study scientists stressed that, at best, wastewater data indicated trends and not absolute numbers of infections. Local health department officials could not foresee incorporating wastewater surveillance into daily practice without a high level of evidence and guidance supporting its adoption.

Several stakeholders mentioned the importance of goal setting between LTCF leadership and the study team. Scientists expressed a desire to define ideal parameters such as target sensitivity and PPV, turnaround time, and methods of communication. LTCF leadership prioritized a streamlined approach to wastewater surveillance, such as an *industry build out* with established resources to easily set up and maintain systems *off the shelf*, such as commercially available fire protection systems.

## Discussion

### Principal Findings

Our evaluation of wastewater surveillance for SARS-CoV-2 at LTCFs found the approach feasible and timely, with results available to stakeholders within approximately 24 to 72 hours. Wastewater surveillance demonstrated decent specificity but poor sensitivity for identifying known SARS-CoV-2 clinical cases, although sensitivity improved when using only clinical test results from LTCF residents (excluding staff). LTCF wastewater surveillance was stable; solutions to environmental challenges were manageable, and there were minimal disruptions in sampling.

Stakeholders consistently raised questions about wastewater surveillance performance and results interpretation. The limitations of clinical testing data, such as less frequent testing of residents and the use of rapid antigen tests, affected the team’s ability to accurately estimate the performance of wastewater surveillance (ie, there was not a gold standard on which to base sensitivity and specificity calculations). Uncertainties also existed regarding the population under wastewater surveillance. Family visits resumed during the surveillance period, staff bowel habits were unknown, and residents with fecal incontinence used adult briefs, meaning that their feces did not enter the wastewater stream. Staff with COVID-19 isolated at home for a variable period before returning to work and potentially contributing to the wastewater stream. These limitations eroded confidence in wastewater surveillance performance.

Stakeholders wanted to quantify the number of COVID-19 cases represented by a specific wastewater signal intensity. The relationship between wastewater signal intensity and number of cases was not clear, owing in part to uncertainties regarding the population under surveillance described in the preceding paragraph. In addition, viral shedding of SARS-CoV-2 is variable in frequency, intensity, and duration [16]. These parameters likely vary among SARS-CoV-2 variants and across host characteristics (age, disease intensity, and comorbid conditions) [17]. Additional facility and environmental considerations may have affected measured wastewater SARS-CoV-2 concentrations, such as variations in wastewater flow, presence of inhibitor substances (cleaning agents and disinfectants), ambient temperature, and the heterogeneous nature of wastewater [18,19]. However, water quality differences among facilities may not correlate with changes in SARS-CoV-2 concentrations [20]. Because of these factors, precisely delimiting the relationship between wastewater SARS-CoV-2 concentration and COVID-19 cases was difficult and may not be realistic at the facility level. Furthermore, once there is an established case in a building, identifying new infections using wastewater measurements remains a hurdle because convalescing individuals continue to shed virus [21]. SARS-CoV-2 RNA present in wastewater from convalescing individuals may decrease the estimated specificity and PPV of the wastewater signal for detecting new SARS-CoV-2 infections.

Variability in turnaround time from sample collection to result dissemination was primarily because of staffing and the day of sample collection. Samples collected on Tuesday through Thursday underwent analysis the same day, with results typically reported the next morning, whereas samples collected on Friday had delayed reporting of results owing to limited weekend staffing. Turnaround times observed in this evaluation were comparable with those reported by a wastewater laboratory in Berkeley, California [22]. Wastewater testing results turnaround time, regardless of the day of the week, was quicker than typical turnaround times for PCR clinical testing (48-72 h) but slower than rapid antigen tests (15 min to 1 h) [7]. Adding staff in the evening or during the weekend could mitigate the observed variability in results turnaround times and support wastewater surveillance as an effective early warning system.

Wastewater surveillance at LTCFs may provide an early warning of infection at a facility before detection by routine screening practices as suggested by the time-shifted correlation analysis. A positive wastewater signal could trigger enhanced clinical testing and infection prevention actions. To maximize the potential lead time provided by the wastewater signal, an LTCF would need clear operating procedures in place. These include capacity to test, perform contact tracing, and isolate individuals who have been potentially infected, as demonstrated on university campuses that used wastewater surveillance to trigger disease mitigation responses [23-25]. Uncertainties in how to interpret the wastewater data and how best to communicate this information within our academic-private partnership limited actions taken in response to wastewater data and require additional investigation in how best to use wastewater surveillance as an infection-detection modality.

Facility wastewater surveillance was flexible, as demonstrated by the rapid resumption of surveillance at the onset of the SARS-CoV-2 Omicron variant wave in January 2022; however, there are structural limitations to this surveillance. Sewer architecture is an important consideration when evaluating facility suitability for wastewater surveillance [26]. Situations where facility-level wastewater surveillance is less suitable include facilities where sewer access is limited to manholes in busy streets, there is low effluent flow, and convergent wastewater streams prohibit the selective sampling of wastewater from the facility of interest. Labor availability or lack of access to a laboratory skilled in RT-qPCR might prolong results turnaround time to a point where wastewater surveillance may not provide an early warning of disease [25].

Most stakeholders expressed enthusiasm for wastewater surveillance, recognizing its cost-effective noninvasive nature and potential for monitoring SARS-CoV-2 variants of concern and other pathogens, including methicillin-resistant *Staphylococcus aureus* and *C difficile*, as well as viruses that cause respiratory infections such as influenza. The specific use cases for wastewater surveillance will vary based on stakeholder priorities, institutional wastewater infrastructure, and available resources. Wastewater surveillance may also serve purposes beyond disease surveillance, such as maintaining relationships with external stakeholders [27]. In our case, the leadership at the LTCF organization were interested in wastewater surveillance as an emerging technology that could protect the well-being of their residents and demonstrate their commitment as an industry leader in safety.

Stakeholders in the LTCF industry cited the lack of easily deployable wastewater surveillance systems as a major hurdle to wider adoption. They described a desire for *off-the-shelf, ready-to-go* wastewater surveillance systems, akin to fire protection systems. The COVID-19 pandemic is driving rapid innovation of wastewater surveillance technology, and as the industry develops and technology improves, LTCF managers may be open to broader adoption of this tool. Alternatively, LTCFs could partner with local utilities or health departments to build sustainable wastewater surveillance systems.

Although reports describing the use of wastewater surveillance at LTCFs are sparse, there are other facility-based settings where wastewater surveillance flourished during the COVID-19 pandemic. Universities, in particular, were early adopters and implemented building-level wastewater testing, while developing wastewater sampling strategies, laboratory assays, and public health response measures [25]. Schools were another congregate setting where wastewater surveillance demonstrated utility in identifying and potentially mitigating SARS-CoV-2 infection [28,29]. Correctional settings have also used and evaluated wastewater surveillance [30], and former inmates have voiced a preference for wastewater surveillance over individual testing for SARS-CoV-2 [31]. Many of the lessons learned from this evaluation of wastewater surveillance at LTCFs may apply to wastewater surveillance in other facility-level settings.

This surveillance evaluation had limitations in addition to the challenges we have already described. Sparse data from relatively few positive clinical tests during the surveillance period reduced the power of the statistical analyses and may have affected surveillance performance estimates. The stakeholders interviewed for the evaluation shared perspectives that may not represent the opinions and experiences of individuals involved in wastewater surveillance in other settings, meaning that the findings from this evaluation may not generalize to other LTCF-based wastewater surveillance settings. However, by adhering to the CDC guidelines for evaluating public health surveillance systems, we used reproducible methods and provide credible data and insight into the performance of wastewater surveillance for SARS-CoV-2 at 6 LTCFs in Kentucky.

### Conclusions and Recommendations

Facility-level wastewater surveillance can monitor populations considered vulnerable for the presence of infectious diseases. Stakeholders found the surveillance feasible and expressed optimism regarding its potential while also recognizing challenges in interpreting, and acting on, the data. Further studies of the performance of facility-level wastewater surveillance will improve the interpretation of wastewater data and increase the utility of this emerging surveillance modality.

Specific recommendations based on this evaluation are to (1) investigate the relationship between wastewater SARS-CoV-2 RNA levels and the number of individuals infected and convalescing at a facility (fecal shedding studies may help elucidate this relationship); (2) synthesize facility-based wastewater surveillance data across projects to develop and test guidance on data interpretation; and (3) foster relationships among academic partners, LTCF organizations, and public health officials to clarify and strengthen communication practices to understand the priorities and limitations of wastewater surveillance.

## Appendix

Multimedia Appendix 1 Key informant semistructured interview guides.

## Abbreviations

CDC: Centers for Disease Control and Prevention
LTCF: long-term care facility
PCR: polymerase chain reaction
PPV: positive predictive value
REDCap: Research Electronic Data Capture
RT-qPCR: real-time qualitative polymerase chain reaction

## Data Availability

The wastewater and interview data evaluated for this study are available from the corresponding author on reasonable request. Clinical test data are available on the Trilogy COVID-19 dashboard [32].

## References

[1] Lau-Ng R, Caruso LB, Perls TT (2020). COVID-19 deaths in long-term care facilities: a critical piece of the pandemic puzzle. J Am Geriatr Soc.

[2] Chidambaram P (2022). Over 200,000 residents and staff in long-term care facilities have died from COVID-19. Kaiser Family Foundation.

[3] Arons MM, Hatfield KM, Reddy SC, Kimball A, James A, Jacobs JR, Taylor J, Spicer K, Bardossy AC, Oakley LP, Tanwar S, Dyal JW, Harney J, Chisty Z, Bell JM, Methner M, Paul P, Carlson CM, McLaughlin HP, Thornburg N, Tong S, Tamin A, Tao Y, Uehara A, Harcourt J, Clark S, Brostrom-Smith C, Page LC, Kay M, Lewis J, Montgomery P, Stone ND, Clark TA, Honein MA, Duchin JS, Jernigan JA, Public Health–Seattle and King County and CDC COVID-19 Investigation Team (2020). Presymptomatic SARS-CoV-2 infections and transmission in a skilled nursing facility. N Engl J Med.

[4] Orive G, Lertxundi U, Barcelo D (2020). Early SARS-CoV-2 outbreak detection by sewage-based epidemiology. Sci Total Environ.

[5] Chen Y, Chen L, Deng Q, Zhang G, Wu K, Ni L, Yang Y, Liu B, Wang W, Wei C, Yang J, Ye G, Cheng Z (2020). The presence of SARS-CoV-2 RNA in the feces of COVID-19 patients. J Med Virol.

[6] Olesen SW, Imakaev M, Duvallet C (2021). Making waves: defining the lead time of wastewater-based epidemiology for COVID-19. Water Res.

[7] Betancourt WQ, Schmitz BW, Innes GK, Prasek SM, Pogreba Brown KM, Stark ER, Foster AR, Sprissler RS, Harris DT, Sherchan SP, Gerba CP, Pepper IL (2021). COVID-19 containment on a college campus via wastewater-based epidemiology, targeted clinical testing and an intervention. Sci Total Environ.

[8] Kirby AE, Walters MS, Jennings WC, Fugitt R, LaCross N, Mattioli M, Marsh ZA, Roberts VA, Mercante JW, Yoder J, Hill VR (2021). Using wastewater surveillance data to support the COVID-19 response - United States, 2020-2021. MMWR Morb Mortal Wkly Rep.

[9] Karthikeyan S, Nguyen A, McDonald D, Zong Y, Ronquillo N, Ren J, Zou J, Farmer S, Humphrey G, Henderson D, Javidi T, Messer K, Anderson C, Schooley R, Martin NK, Knight R (2021). Rapid, large-scale wastewater surveillance and automated reporting system enable early detection of nearly 85% of COVID-19 cases on a university campus. mSystems.

[10] LaJoie AS, Holm RH, Anderson LB, Ness HD, Smith T (2022). Nationwide public perceptions regarding the acceptance of using wastewater for community health monitoring in the United States. PLoS One.

[11] German R, Lee L, Horan J, Milstein RL, Pertowski CA, Waller MN, Guidelines Working Group Centers for Disease Control and Prevention (CDC) (2001). Updated guidelines for evaluating public health surveillance systems: recommendations from the Guidelines Working Group. MMWR Recomm Rep.

[12] Watkins DC (2017). Rapid and rigorous qualitative data analysis: the “RADaR” technique for applied research. Int J Qual Methods.

[13] Berry SM, Pezzi HM, Williams ED, Loeb JM, Guckenberger DJ, Lavanway AJ, Puchalski AA, Kityo CM, Mugyenyi PN, Graziano FM, Beebe DJ (2015). Using exclusion-based sample preparation (ESP) to reduce viral load assay cost. PLoS One.

[14] Strike W, Amirsoleimani A, Olaleye A, Noble A, Lewis K, Faulkner L, Backus S, Lindeman S, Eterovich K, Fraley M, Dehghan Banadaki M, Torabi S, Rockward A, Zeitlow E, Liversedge M, Keck J, Berry S (2022). Development and validation of a simplified method for analysis of SARS-CoV-2 RNA in university dormitories. ACS ES T Water.

[15] Oran DP, Topol EJ (2021). The proportion of SARS-CoV-2 infections that are asymptomatic: a systematic review. Ann Intern Med.

[16] Walsh KA, Jordan K, Clyne B, Rohde D, Drummond L, Byrne P, Ahern S, Carty PG, O'Brien KK, O'Murchu E, O'Neill M, Smith SM, Ryan M, Harrington P (2020). SARS-CoV-2 detection, viral load and infectivity over the course of an infection. J Infect.

[17] Cevik M, Tate M, Lloyd O, Maraolo AE, Schafers J, Ho A (2021). SARS-CoV-2, SARS-CoV, and MERS-CoV viral load dynamics, duration of viral shedding, and infectiousness: a systematic review and meta-analysis. Lancet Microbe.

[18] Buonerba A, Corpuz MV, Ballesteros F, Choo KH, Hasan SW, Korshin GV, Belgiorno V, Barceló D, Naddeo V (2021). Coronavirus in water media: analysis, fate, disinfection and epidemiological applications. J Hazard Mater.

[19] Kitajima M, Ahmed W, Bibby K, Carducci A, Gerba CP, Hamilton KA, Haramoto E, Rose JB (2020). SARS-CoV-2 in wastewater: state of the knowledge and research needs. Sci Total Environ.

[20] Sharkey ME, Kumar N, Mantero AM, Babler KM, Boone MM, Cardentey Y, Cortizas EM, Grills GS, Herrin J, Kemper JM, Kenney R, Kobetz E, Laine J, Lamar WE, Mader CC, Mason CE, Quintero AZ, Reding BD, Roca MA, Ryon K, Solle NS, Schürer SC, Shukla B, Stevenson M, Stone T, Tallon JJ Jr, Venkatapuram SS, Vidovic D, Williams SL, Young B, Solo-Gabriele HM (2021). Lessons learned from SARS-CoV-2 measurements in wastewater. Sci Total Environ.

[21] Colosi LM, Barry KE, Kotay SM, Porter MD, Poulter MD, Ratliff C, Simmons W, Steinberg LI, Wilson DD, Morse R, Zmick P, Mathers AJ (2021). Development of wastewater pooled surveillance of severe acute respiratory syndrome coronavirus 2 (SARS-CoV-2) from congregate living settings. Appl Environ Microbiol.

[22] Kantor RS, Greenwald HD, Kennedy LC, Hinkle A, Harris-Lovett S, Metzger M, Thornton MM, Paluba JM, Nelson KL (2022). Operationalizing a routine wastewater monitoring laboratory for SARS-CoV-2. PLoS Water.

[23] Scott LC, Aubee A, Babahaji L, Vigil K, Tims S, Aw TG (2021). Targeted wastewater surveillance of SARS-CoV-2 on a university campus for COVID-19 outbreak detection and mitigation. Environ Res.

[24] Gibas C, Lambirth K, Mittal N, Juel MA, Barua VB, Roppolo Brazell L, Hinton K, Lontai J, Stark N, Young I, Quach C, Russ M, Kauer J, Nicolosi B, Chen D, Akella S, Tang W, Schlueter J, Munir M (2021). Implementing building-level SARS-CoV-2 wastewater surveillance on a university campus. Sci Total Environ.

[25] Harris-Lovett S, Nelson KL, Beamer P, Bischel HN, Bivins A, Bruder A, Butler C, Camenisch TD, De Long SK, Karthikeyan S, Larsen DA, Meierdiercks K, Mouser PJ, Pagsuyoin S, Prasek SM, Radniecki TS, Ram JL, Roper DK, Safford H, Sherchan SP, Shuster W, Stalder T, Wheeler RT, Korfmacher KS (2021). Wastewater surveillance for SARS-CoV-2 on college campuses: initial efforts, lessons learned, and research needs. Int J Environ Res Public Health.

[26] Reeves K, Liebig J, Feula A, Saldi T, Lasda E, Johnson W, Lilienfeld J, Maggi J, Pulley K, Wilkerson PJ, Real B, Zak G, Davis J, Fink M, Gonzales P, Hager C, Ozeroff C, Tat K, Alkire M, Butler C, Coe E, Darby J, Freeman N, Heuer H, Jones JR, Karr M, Key S, Maxwell K, Nelson L, Saldana E, Shea R, Salveson L, Tomlinson K, Vargas-Barriga J, Vigil B, Brisson G, Parker R, Leinwand LA, Bjorkman K, Mansfeldt C (2021). High-resolution within-sewer SARS-CoV-2 surveillance facilitates informed intervention. Water Res.

[27] Harris-Lovett S, Nelson KL, Kantor R, Korfmacher KS (2023). Wastewater surveillance to inform public health decision making in residential institutions. J Public Health Manag Pract.

[28] Hassard F, Vu M, Rahimzadeh S, Castro-Gutierrez V, Stanton I, Burczynska B, Wildeboer D, Baio G, Brown MR, Garelick H, Hofman J, Kasprzyk-Hordern B, Majeed A, Priest S, Denise H, Khalifa M, Bassano I, Wade MJ, Grimsley J, Lundy L, Singer AC, Di Cesare M (2023). Wastewater monitoring for detection of public health markers during the COVID-19 pandemic: near-source monitoring of schools in England over an academic year. PLoS One.

[29] Wolken M, Sun T, McCall C, Schneider R, Caton K, Hundley C, Hopkins L, Ensor K, Domakonda K, Kalvapalle P, Persse D, Williams S, Stadler LB (2023). Wastewater surveillance of SARS-CoV-2 and influenza in preK-12 schools shows school, community, and citywide infections. Water Res.

[30] Klevens RM, Young CC, Olesen SW, Osinski A, Church D, Muten J, Chou L, Segal T, Cranston K (2023). Evaluation of wastewater surveillance for SARS-CoV-2 in Massachusetts correctional facilities, 2020–2022. Front Water.

[31] Riback LR, Dickson P, Ralph K, Saber LB, Devine R, Pett LA, Clausen AJ, Pluznik JA, Bowden CJ, Sarrett JC, Wurcel AG, Phillips VL, Spaulding AC, Akiyama MJ (2023). Coping with COVID in corrections: a qualitative study among the recently incarcerated on infection control and the acceptability of wastewater-based surveillance. Health Justice.

[32] Trilogy Health Services.

