# Supplementary material for: Wastewater Surveillance for SARS-CoV-2 at Long-Term Care Facilities: Mixed Methods Evaluation
Source: JMIR Public Health Surveill. 2023 Aug 29;9:e44657. doi: 10.2196/44657 (PMC10467632; doi:10.2196/44657)
Supplement: Multimedia Appendix 1 [file publichealth_v9i1e44657_app1.docx]

**Multimedia Appendix 1**

**Key Informant Semi-Structured Interview Guide**

**Background: I am part of a team that is studying wastewater surveillance for COVID-19 virus as a tool to identify and monitor infections in nursing home populations. We are evaluating the performance of nursing home wastewater surveillance and are speaking with various stakeholders to help guide our evaluation. We want our evaluation to be responsive to your concerns and needs. With your permission, I will record our conversation and take notes so I can review your responses at a later time.**

**Long-term care facility staff and leadership questions:**

[pre interview] Obtain informed consent. Share that the interview will be recorded for later review.

Q1. Please describe your role and responsibilities at [insert facility/organization name].

Q2. What are your primary sources of information to monitor infection risk at your facility?

Q3. What is your understanding of wastewater surveillance for SARS-CoV-2? What do you think about it?

Q4. Has your facility or organization used wastewater surveillance for SARS-CoV-2? (IF NO – BRANCH TO Q9)

Q5. How has your facility or organization used the results of wastewater surveillance? [potential probes: infection prevention measures, resident/staff testing, risk communication] → Has the way you used the results changed over time?

Q6. What resources has your facility or organization used to support the wastewater surveillance? [potential probes: personnel, time commitments, infrastructure, consumables]

Q7. Have there been any challenges or problems with the wastewater surveillance? If yes, what? [potential probes: timeliness, communication, interpretation of results, resource burden]

Q8. What might ideal communication of wastewater surveillance data look like? [potential probes: frequency of communication, platform or method of communication, raw data, figures/ graphics, shared files]

Q9. How could wastewater surveillance be improved to be more useful for your facility?

Q10. Would you consider using wastewater surveillance as part of your infection risk management strategy? Why or why not?

Q11. What are your biggest concerns about using this type of information?

**CDC subject matter expert questions:**

[pre-interview] Obtain informed consent. Share that the interview will be recorded for later review.

Q1. As a CDC subject matter expert, how do you view your role in this wastewater surveillance for SARS-CoV-2 project?

Q2. How do you interact with wastewater surveillance for SARS-CoV-2 data in your role at CDC?

Q3. What attributes of facility-level wastewater surveillance are important to understand?

Q4. What else is important to know about nursing home wastewater? [potential probes: costs, resources needed, communication or interpretation of results]

**Lab and field staff additional questions:**

[pre-interview] Obtain informed consent. Share that the interview will be recorded for later review.

Q1. What is your role in the project?

Q2. What challenges have you encountered during this project? [potential probes: coordination with remote partners, process issues, communication, resources, technology, environmental factors]

Q3. What are some important lessons learned regarding wastewater surveillance for SARS-CoV-2?

What do you want to learn from an evaluation of this project?

Q4. (Research team): For generalizing this project to other nursing homes/ facilities- could you please describe the average amount of time per week needed for your role?

Q5. (Research team): What resources were essential to complete your role?

**State/local health official additional questions:**

[pre-interview] Obtain informed consent. Share that the interview will be recorded for later review.

Q1. What is your role at the state or local health department?

Q2. Have you used, or do you use, wastewater surveillance data in your role? If not, why not?

Q3. What sources and types of SARS-CoV-2 data do you use?

Q4. How do you act on or communicate your findings/ guidance to nursing home leadership? To the public?

Q5. What are your concerns regarding wastewater surveillance for SARS-CoV-2? Do you have special concerns considering wastewater surveillance at a facility level for nursing homes?

Q6. What questions about wastewater surveillance should we try to answer with our evaluation?
